# Supplementary material for: A High Performance Polyacrylonitrile Composite Separator with Cellulose Acetate and Nano-Hydroxyapatite for Lithium-Ion Batteries
Source: Membranes (Basel). 2022 Jan 20;12(2):124. doi: 10.3390/membranes12020124 (PMC8880128; doi:10.3390/membranes12020124)
Supplement: Supplementary file 1 [file membranes-12-00124-s001.zip › membranes-1552715-supplementary.pdf]

membranes

Supplementary information

With increasing time, the contact angle of PAN/CA/HAP-1.0 separator at different times of the electrolyte becomes progressively smaller.

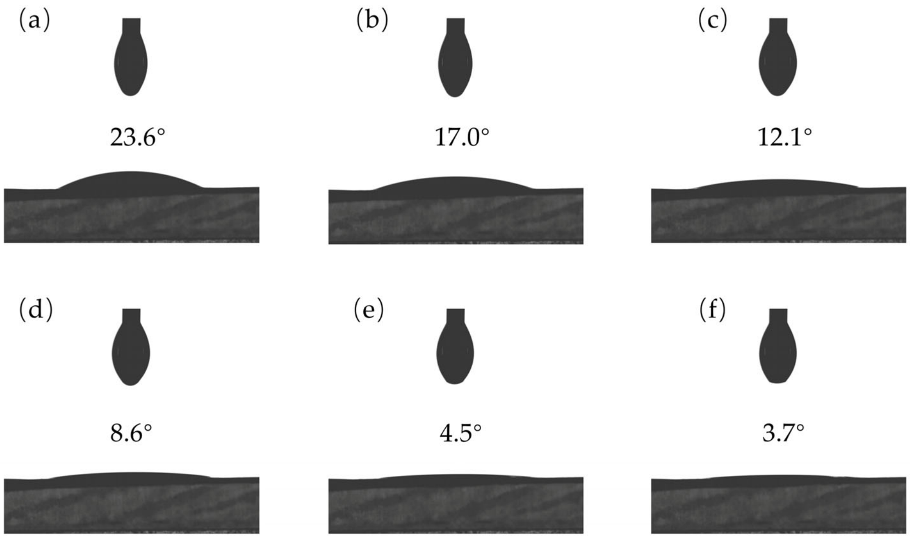

Figure S1: The contact angles of PAN/CA/HAP-1.0 separators with electrolyte at different times. (**a**) 0 s, (**b**) 0.33 s, (**c**) 2.97 s, (**d**) 9.70 s, (**e**) 21.53 s, (**f**) 28.13 s.
